# The National Bureau of Standards and the Radium Dial Painters

**DOI:** 10.6028/jres.126.051

**Published:** 2022-02-14

**Authors:** Bert M. Coursey

**Affiliations:** 1 Standards Coordination Office, National Institute of Standards and Technology, Gaithersburg, MD 20899, USA

**Keywords:** electroscopes, radium-226 measurements, radium dial painters, radium poisoning, radon, U.S. Bureau of Standards

## Abstract

The tragedy of the radium poisoning of young women dial painters in the 1920s has been the subject of best-selling books, plays, and motion pictures. With knowledge about radium and its accurate measurements in the hands of a very few scientists, what responsibilities did they have to sound the alarm and mitigate the hazards to workers and the general public? This two-part analysis looks at the role of the staff of the U.S. Bureau of Standards (the National Bureau of Standards [NBS] after 1934) in developing measurements and standards for accurate determinations of radium-226 and radon-222 that ultimately led to national standards for exposure to radioactive substances. Part I looks at the efforts of Elizabeth Hughes, with guidance from her senior colleague at the NBS, to assist dial painters with obtaining redress for their injuries. Part II examines the role of NBS in establishing the national radiation protection standards that were promulgated by the U.S. Department of Commerce (DOC) and the National Council on Radiation Protection and Measurements (NCRP).

## Part I: Elizabeth E. Hughes and Noah Ernest Dorsey

## 1 Introduction

Elizabeth Elmore Hughes (née Damon), a 30 year old woman with a baccalaureate degree in general science, was one of the leading U.S. scientists in the 1928 trial involving the radium dial painters against the U.S. Radium Corporation [1, 2]. Her initiative and her training as a laboratory assistant at the U.S. Bureau of Standards^1^1 The official title of the institute in 1920 was the U.S. Bureau of Standards. It was renamed the National Bureau of Standards in 1934 and the National Institute of Standards and Technology in 1987. For consistency, we refer throughout to the National Bureau of Standards (NBS). allowed her to inform the Chancery Court of New Jersey on the physics and chemistry of radium and radon, and the details of her radium measurements of the young women who suffered serious injuries and early deaths.

Hughes (Damon at the time) attended the University of Rhode Island and then graduated from the University of Vermont in 1919 with studies in physics, chemistry, and mathematics [1] (Appendix A). She then took a position as a laboratory assistant in the Radium Section at NBS. The title is significant because the section at the time identified staff as aids, laboratory assistants, and assistant physicists. Samuel Wesley Stratton, the director at the time, had to be pressured to hire women at the bureau [3]. The Radium Section in 1920 had one physicist, Section Chief Noah Ernest Dorsey, one assistant physicist, Walter Hiram Wadleigh, six laboratory assistants, and four aids [4]. In his annual report in 1920, Dorsey complained about the frequent turnover of staff and the high expectations for the junior workers [4]:

“In the radium work it is desirable to employ Aids of more than usual ability. Such an aid regards his position at the Bureau as merely temporary, to be held while he is studying, acquiring experience, or looking for a position elsewhere. The same is true of most of the Laboratory Assistants of ability.”

Hughes must have been recognized for her abilities because she was assigned the important task of calibrating sealed radium sources using the gold-leaf electroscope that Dorsey described in his 1921 book *Physics of Radioactivity* [5] (Appendix B).

On June 30, 1920, Dorsey took a leave of absence to work on said textbook. After six and a half years of working with gram quantities of radium, he was literally burned out, mentally and physically. He said as much in a resignation letter written in April 1920 to his long-time supervisor, Chief Physicist Edward Bennett Rosa. Dorsey returned to NBS a few years later and worked for many years in other applied physics programs [4]. Hughes left the NBS a month later to take a position as a physicist at the Radium Luminous Materials Corporation (RLMC) in Orange, NJ. She was likely recruited because of her experience in the NBS Radium Section, and possibly a recommendation from Dorsey. She noted in court testimony eight years later that she had always been in contact with Dorsey [2].

The RLMC was founded in 1914 by two New York physicians, Sabin Albin von Sochocky and George S. Willis [6]. von Sochocky (1883–1928) was a Ukrainian who had studied physics and chemistry at the Lviv University in the Ukraine and obtained his degree in medicine at the University of Moscow (Appendix C). In 1906, he studied radioactivity with the Curies in Paris and came to New York to practice medicine. In 1913, he produced for commercial use a fluorescent paint by mixing small amounts of radium-226 with zinc sulfide and other additives.^2^2 The paint used by the dial painters in the New Jersey factory contained chiefly zinc sulphide rendered luminous by activation with about 1 mg of radium element (or its equivalent in mesothorium) to 30 – 40 g of zinc sulfide. The proprietary formulations of the zinc sulphide contained trace amounts of other elements to increase the luminosity: cadmium (0.05%, copper (0.001%), and manganese (0.0002%) [7]. The alpha particles from the radium decay excited the zinc sulfide to provide a continuous light source. This discovery quickly led to a lucrative industry that provided luminous dials and signage for ships and cockpits for the U.S. Navy and Army in World War I [8, 9, 10, 11]. The industry also developed a commercial market for luminous-dial watches and other products [12]. By 1917, the RLMC complex in Orange, NJ, consisted of several buildings [13] devoted to extracting the radium from carnotite ores (high in uranium and radium content) from the Paradox Valley region in Colorado. von Sochocky, in his role as the chief scientist, supervised extraction and refinement of about 30 g of radium by 1921 [14, 15]. The extraction process that they developed led to production of gram quantities of radium, which rendered the plant itself totally unsuitable for assaying small quantities of samples with the electroscope. Accurate measurements of samples with micrograms of activity were required to optimize radium extraction chemistry. von Sochocky himself was so contaminated with radium that any measurements that he made with the electroscope had to be corrected for his excess background [6, 7, 13]. Florence Wall, a chemist who came to RLMC in 1917, described the conditions in the plant and problems with the radioactivity measurements using the electroscopes. She left after one year. Elizabeth Hughes arrived in August 1920 to work in the Electroscope Laboratory, which was 1.2 km (¾ miles) away from the extraction plant [1, 13].

In court testimony in 1928, Hughes described her work as a physicist [2]:

“Examination of the ore through crystallization; examination of the ore, examinations of the solutions containing radium through crystallization process and final determination of the radium salts.”

**Fig. 1:**
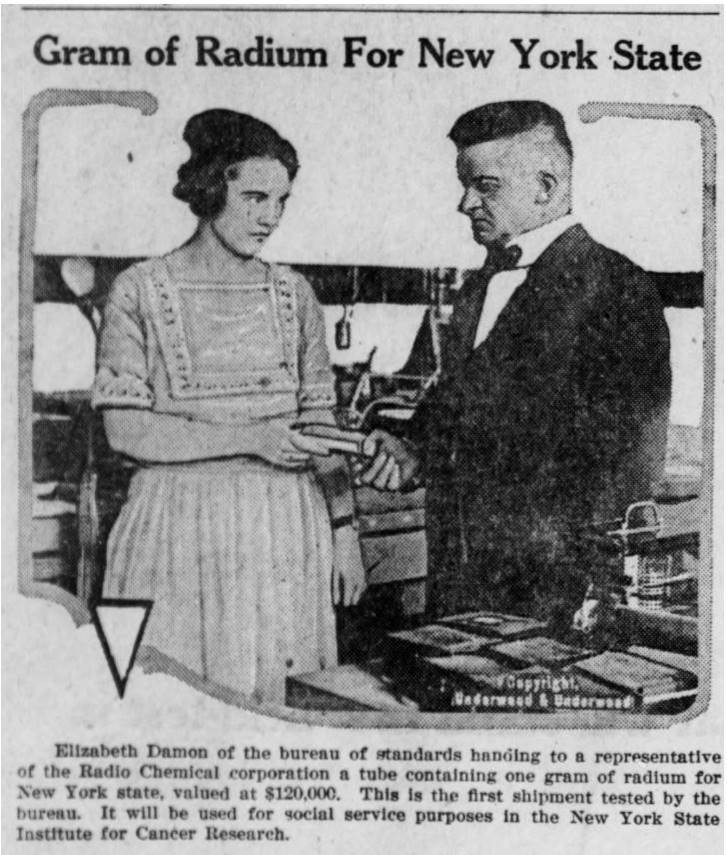
Elizabeth Damon Hughes from U.S. Bureau of Standards handing a radium standard to a customer from U.S. industry [16]. Fulton County Tribune, August 13, 1920, Wauseon, OH. https://chroniclingamerica.loc.gov/lccn/sn87076552/1920-08-13/ed-1/seq-7/

She said this was done for plant control. Her measurements were clearly used by the chemist Edwin Leman and RLMC President von Sochocky to optimize the steps in the extraction processes from the time the ore arrived until the final radium sulfide was packaged for use with the luminous paint. When she arrived in 1920, she did not have any direct supervision in the laboratory. Her results were transmitted by a messenger boy to the plant. She reported that in her previous position at NBS, she had worked under Dr. Dorsey on gamma-ray determination of all radium preparations sold in the United States. The purpose of her work at NBS was “to determine the amount of radium that the different companies were selling to doctors.” She is shown in a newspaper photograph (Fig. 1) in August of 1920 [16] handing a container said to contain a gram of radium to a representative of the “Radio Chemical corporation.” This is probably the Radium Chemical Company of New York, a subsidiary of the Standard Chemical Company of Pittsburgh [15, 17].

Here, it is useful to look at the state of the radium industry in the United States at the time. Edward Landa from the U.S. Geological Survey has done extensive research on the early radium industry in the United States [15]. In addition to the RLMC, there were a few other mining and radium extraction companies [17]. The largest and most successful of these was the Standard Chemical Company of Pittsburgh. They used carnotite ores also from Paradox Valley mines and claims and separated the radium at a plant in Canonsburg, PA. The Standard Chemical Company also established a subsidiary, the Radium Dial Company, to prepare luminous signs at a plant in Illinois. A third major player was the Radium Company of Colorado in Denver. All three radium producers used carnotite ores from southwestern Colorado and adjacent regions of eastern Utah. Their customers were the military for luminous signage, major watch manufacturers, and the rapidly expanding medical profession for radium therapy. Landa reports in 1920, for example, that the United States produced 32.5 grams of radium, of which the Standard Chemical Company alone accounted for 18.5 grams [15]. The NBS reported for the fiscal year July 1, 1919, to June 30, 1920, that they performed in excess of 1400 calibrations of a total of 25.5 grams of radium. It seems reasonable to accept the claims from NBS at the time (and Elizabeth Hughes later) that the Radium Section calibrated all of the radium sold for medical use, and they probably validated the assays for commercial shipments provided to the U.S. Navy and Army [4].

Mesothorium, or radium-228, which is a decay product of natural thorium-232, was also in use at the time in parallel applications. The commercial source of the 5.75 year half-life radium-228 was monazite containing high concentrations of thorium. The Welsbach Company in Gloucester City, NJ, across the river from Philadelphia, extracted the thorium for use in gas-lighting mantles [15]. The chemist Herman Schlundt (1869–1937) was a specialist on mesothorium [18, 19]. Schlundt worked for the U.S. Bureau of Mines (USBM) in Denver and then became a professor of chemistry at the University of Missouri [20]. He was introduced by a USBM colleague, Samuel Colville Lind (1879–1965), to officials at the Welsbach Company. Schlundt then developed the chemistry for extraction of the mesothorium from wastes generated at the Welsbach plant. For 12 years, Schlundt served as a consultant to the Welsbach Company. The radium-228 extracted was shipped 145 km (90 miles) north to the RLMC site in Orange, NJ, for use in luminous dial paints. In 1921, Schlundt spent a year at the Cavendish Laboratory in Cambridge, U.K., with Ernest Rutherford [20]. Both Schlundt and his friend Lind figured prominently in the case of the dial painters later in the 1920s.

For her first year at the RLMC, Hughes was on her own in the Electroscope Laboratory. However, after World War I ended, the demand for signage for the military dropped, and the company was reorganized. von Sochocky was replaced by RLMC Treasurer Arthur Roeder, and the new company was established as the U.S. Radium Corporation (USRC) [10, 11]. In 1921, the USRC recruited a renowned European physicist, Victor Hess (1883–1964). Hess, an Austrian, had discovered cosmic rays in 1912 and had worked with Stefan Meyer, the secretary of the International Radium Standards Commission at the Institute for Radium Research in Vienna [21]. Hughes reported to Hess for the following year. She had positive remarks about Hess and his understanding of the physics of radioactivity. He included her as a coauthor on a 1922 paper in *Physical Review* on rapid methods for measuring radium in ores [22]. Hughes had worked with the Lind electroscope in her time at the NBS [2]. During his two years in the United States, Hess consulted with the USBM in Washington, D.C., as well as the USRC, so it is highly likely that he worked with Herman Schlundt, who was consultant to the USRC, and with Samuel Lind at the USBM. He returned to Austria in 1923 to take a position in physics at the University of Graz. Hess would receive the 1936 Nobel Prize in Physics for his discovery of cosmic rays [21].

During the years that Hughes was making quality-control measurements for radium in New Jersey, the radium metrology program at NBS was undergoing significant disruptions and changes in direction. When Dorsey left in 1920, the Radium Section was led by Walter Hiram Wadleigh (1873–1968), a physicist who had come from the University of Arkansas during World War I [4]. The high point of the NBS radium program came in May of 1921 when Marie Curie visited Washington, D.C., to receive a gram of radium from President Harding at the White House [23]. The 10 ampoules containing the 1 g of radium were prepared by the Standard Chemical Company of Pittsburgh and measured at NBS against the U.S. International Secondary Standard Number 6, the 15.44 mg source that had been certified by Marie Curie, Ernest Rutherford, and Stefan Meyer. The NBS Director Samuel Wesley Stratton was on the local organizing committee for Curie’s visit to Washington, D.C., and arranged her visits to NBS. On Sunday, May 22, 1921, she was accompanied by her daughter Irene on a visit to NBS [24]. She probably discussed the radium measurements with Wadleigh, who later took the box containing the radium to the sailing ship *Olympic* in New York for the Curies’ return trip to France [25]. Wadleigh made no significant contributions to the radium metrology program, and, when he left in 1923, he took away some of the valuable records from Curie’s visit, which were returned to NBS by his daughter in 1968 [4]. In 1924, the Radium Section was incorporated into the Atomic Physics, Radium and X-Ray Section in Division IV Optics under the spectroscopist Paul D. Foote (1888–1971).

There were two important additions to the staff for the radioactivity work during that period. In 1920, Constance Torrey (1899–1949), a recent physics graduate from Smith College, arrived to take up the radium source calibration work. Elizabeth Hughes and Torrey did not overlap in 1920. For the next 30 years, Torrey had significant responsibilities for the radium calibrations and signed some of the correspondence with foreign customers. (All certificates were signed by or for the NBS director.) The second key staff member was Leon Francis Curtiss, who arrived in 1926, having spent the past four years on a fellowship at the Cavendish Laboratory with Ernest Rutherford. Curtiss, a physicist from Cornell University, spent the next 35 years at NBS developing a suite of instruments and techniques for radionuclide metrology. He had the opportunity to pursue basic research on decay schemes for radium daughter radionuclides and to begin a decades-long project on cosmic rays [26].

## 2 The “Radium Girls”

The tragedy of the poisoning of young women, the radium dial painters, in New Jersey, Illinois, and Ottawa, Canada, has been thoroughly detailed in three books: *Radium Girls: Women and Industrial Health Reform, 1910–1935*, published in 1997 by Claudia Clark [8, 9]; Ross Mullner’s *Deadly Glow: The Radium Dial Worker Tragedy*, published in 1999 [10]; and Kate Moore’s *Radium Girls: The Dark Story of America’s Shining Women*, published in 2017 [11]. A recent important contribution is an excellent retrospective dosimetry study of the early radium workers by Martinez *et al*. [12]. There are overlapping themes that make the case of the young radium dial painters such a compelling story. First, the 1920s witnessed at least the beginning of an effective public health service in the United States. Second, there was greater public awareness and concern about occupational health hazards (*e.g.*, mercury, phosphorus, and leaded gasoline). Third, as a consequence of the women’s suffrage movement of the 1920s, more women were willing to speak out on behalf of women in the workplace. Against the backdrop of all these societal changes taking place in the nation, a few dozen young women began to show symptoms of serious illness. The causality between their work with small amounts of radium and the deleterious health effects was not obvious at first. Corporate managers, aided and abetted by academic scientists, public health officials, and physicians, first denied the connection between ingestion of radium and dental problems, radium-jaw, and anemia that came from repeated exposures [8, 9].

Exposure to large external sources of radium, such as the milligram sources used in cancer therapy, was of course known to be hazardous. Hughes noted that she was instructed on the hazards of radium by Dorsey from the time she arrived at NBS in 1919 [2]. She said that this was common knowledge to those who read Rutherford’s book on radioactivity [27, 28]. Furthermore, she was personally aware of the radium burns Dorsey had suffered. They both left the NBS in the summer of 1920. In January 1922, the fledgling U.S. Public Health Service (USPHS), with assistance from the new NBS Radium Section Chief Hiram Wadleigh, began an 18 month study of the section’s staff [29]. The purposes of the study were: (1) the necessity of periodic supervision of the physical condition of persons engaged in the constant handling of radium, in order to properly safeguard their health, and (2) as a matter of scientific interest, to note the physical effects upon radiation workers of continued exposure to radiation.

**Fig. 2:**
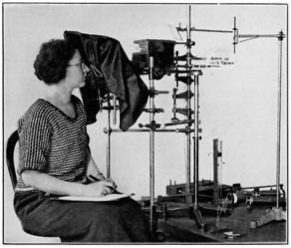
Mary Brower making measurements with the electroscope. *Washington Evening Star*, June 8, 1924 [30].

A detailed report of the study was published in December 1923 by R. C. Williams, assistant surgeon, Office of Industrial Hygiene and Sanitation of the USPHS [29]. The staff of the Radium Section were the perfect cohort on which to carry out such a study because they handled the calibration of almost all the radium sold in the United States at the time, and this group of federal employees was reasonably stable over the 18 months of the observations. It is difficult to imagine such an extensive, controlled study in an operating factory or a therapeutic clinic. The tables delineating the physicians’ observations of the 12 employees over the course of the study do not identify the individuals by name, but it is easy to identify Dorsey and Wadleigh (both born in 1873), the two listed 49 year old males. The report notes that one of these males was a former member who had sustained radium burns to his hands over 6.5 years (equal to the time Dorsey spent working with radium). Mary Brower, a female member of the staff, calibrated radium sources. She is shown here (Fig. 2) in one of the photographs from Williams’ 1923 report [29]. The same photograph was published in 1924 in the *Washington Evening Star* [30]. It seems likely that Torrey and Brower were two of the female staffers included in the USPHS study. Only five of the staff were followed over the entire 18 month study, and that number likely included the four mentioned here.

A full account of the deleterious effects of external whole-body radiation exposure is beyond the scope of the present work, but it is worth noting that the USPHS started with the expectation of finding the following [29]:

“…the now well-recognized blood changes.”

“The polymorphonuclear leucocytic and the lymphocytic blood content of radium workers is decidedly lower than that of normal individuals.”

“The low polymorphonuclear blood content commonly found and the anemia of an aplastic type affecting the most exposed workers point to an interference with the output of blood cells from the bone marrow.”

The detailed physiological parameters for the 12 individuals through the course of the study are given in the USPHS report [29]. Because of expected problems with anemia, blood samples were taken on the staff to identify any changes. Referencing this study, Stewart reported in 1929 [31] regarding the procedures at NBS that,

“All known precautions against radiation are employed, and for several years no indications of injury have been found in the blood of the employees, which is examined regularly every two months.”

The USPHS investigators also designed some experiments in which they placed dental X-ray film on different positions on the body of various workers who routinely handled radium sources [17]. This quickly revealed that the forehead was receiving rather large radiation doses, and they immediately introduced measures to limit doses for the workers. These were possibly some of the first uses of film-badge dosimeters for radiation workers. They also noted that some of the radium sources submitted for calibration were leaking, which resulted in inhalation of radon by the staff. It was recommended that the facility be equipped with electric fans to reduce the exposure to the lungs from the radioactive gas. Although they suspected radiation damage to the bone marrow, they did not make the connection that radium, which is in the same alkaline earth family as calcium, would be taken up in the hydroxy apatite of growing bones and teeth.

The insidious problem of ingested radium was only slowly recognized for several reasons. From the turn of the century, much of the public believed in the therapeutic value of radium. Radiothor and other radon generators were advertised for their beneficial effects [7, 10, 15]. Some of the leading U.S. physicians in cancer therapy experimented with internal administration of radium. For example, Dr. Howard Kelly (1858–1943), whose private practice of radium therapy in Baltimore, MD, in time transitioned to become the department of radiology for Johns Hopkins University Hospitals, initially tried internal administrations [9]. Kelly was one of the partners who founded the National Radium Institute in 1913 [10, 15]. The dial painters in New Jersey were probably in the worst position because the chief scientist advising the USRC, Herman Schlundt, had taken oral administrations of radium [20]. He wanted to see how quickly it would clear his urine. The workers at the Radium Dial Company in Illinois fared no better because Charles Viol, chief chemist, and Frederick Proescher, a medical doctor at the Standard Chemical Company in Pittsburgh (the parent corporation), used their company magazine *Radium* to promote internal uses of radium. The Standard Chemical Company provided “standard” solutions of radium for internal oral and intravenous therapeutic applications. The American Medical Association provided approval for use of these products in 1914 [17] and did not withdraw this approval until 1932 [32].

The 1997, Clark’s book gives an exhaustive account of the public health implications of the case of the dial painters [9]. Mullner’s 1999 book shows the important linkage between the understanding of the dangers of radium and the timely development of national radiation-safety standards for workers on the Manhattan Project [10]. Moore’s book in 2017 takes a closer look at the lives and travails of the individual young women and their families [11]. All three books reflect the extensive research of the authors on the trials and the newspaper accounts of the day. However, the scientific aspects of these investigations were not the primary focus of these accounts. Elizabeth Hughes is mentioned only briefly in Clark’s book as a physicist who had worked at the U.S. Radium Corporation and at the U.S. Bureau of Standards [9].

However, Clark did pose important questions in the preface to her book [9]:

“Two vexing questions posed by the dial painting experience are these: Did researchers studying the dial painters’ illnesses have an ethical duty to publicize the dangers they discovered in workplaces? Did they have an ethical duty to share the knowledge and skills with the dial painters?”

“…with knowledge of the health effects of radium and the skills to measure those effects held by a very small number of scientists, we must consider whether those with knowledge and skills had a greater ethical duty to share them than if such knowledge and skills were widely available.”

This account will look at a small number of U.S. scientists in light of Clark’s questions. What did they know about the science? What experimental tools were available? How did they share their knowledge with the public and with the victims? The books on the dial painters pose these same questions for the physicians and public health officials and identify a few heroes and more than a few villains. To “follow the science” from the perspectives of the chemists and physicists, we need to additionally ask: What did they understand about the chemical and physical properties of radium and radon? What standards were available? What instruments were available and what were the uncertainties associated with their measurements?

## 3 Physics and Chemistry of Radium Isotopes

By 1920, the basic chemistry and physics of uranium, thorium, and the radium isotopes of masses 226 and 228 were fairly well understood. The chemical processes that Marie Curie developed at the turn of the century had been improved on and optimized by industrial chemists at factories (in collaboration with their colleagues in academia) in Europe and the United States. Curie had prepared radium chloride and a small sample of radium metal for her research [33], but the industrial chemists found it easier to separate the radium from the carnotite ore as the bromide [17]. By 1920, gram quantities of radium in sulfuric acid solution were prepared to provide a continuous supply of radium “emanation.” The Colorado physicist William Duane had worked in Curie’s laboratory to optimize the “radium cow,” which was a solution containing radium sulfate that could be “milked” to draw off the radon gas [34]. These radionuclide generators were used to prepare millicurie-level radon-222 sources for use in cancer therapy.

Details of the decay characteristics of uranium-238 and thorium-232 progeny (including radium-226 and radium-228, respectively) were covered well in Rutherford’s 1913 textbook, which he continued to update with additional details. Hughes noted that Rutherford’s books [27, 28] were well known in the United States. The 1600 year half-life radium-226 decay leads quickly to four alpha particles, with a fifth alpha particle from polonium-210 delayed by ingrowth of the parent lead-210. The first decay product is a noble gas (radon-222) initially identified as “radium emanation.” This 3.82 day half-life radon-222 decays by alpha particle emission to shorter-lived daughters. The 5.8 year half-life radium-228 (mesothorium) decay leads to five alpha particles. The second decay product of radium-228 is also a radon isotope (radon-220) identified as “thorium emanation,” or “thoron.” The 55.4 second half-life radon-220 also decays by alpha emission. Both radium isotopes were used in the luminous paints at USRC [15]. The study by Martinez *et al*. provided an overview of the radiobiology of radium and mesothorium, and their daughter products. The study looked at estimated radiation doses and associated radiation health effects [12].

Analytical radium and radon measurements in the 1920s depended on systems that included an electroscope such as the one described by Dorsey in Appendix B. Several different designs were available in the early 1920s. The “gold standard” for radium at the time was the 15.44 mg radium-226 ampoule at NBS that had been certified by the International Radium Standards Commission [23]. The NBS also maintained other working standards in the range from 1 mg to 50 mg such that radioactive specimens could be compared by electroscope with a standard of comparable mass [4]. With working standards that had been calibrated at the NBS (initially by Hughes and later by Brower and Torrey), the commercial firms (USRC and the Standard Chemical Company (SCC)) could also calibrate milligram-level sources with uncertainties that were probably less than 5%. (The SCC provided the first “standards” to Dorsey in 1913, and subsequent comparisons of standards indicated the SCC in-house standards were comparable to those at NBS.) Uncertainties in the assays of less than 5% were required for commercial transactions of the most valuable material on Earth; for perspective, gold was priced at $0.66 per gram compared to $100,000 per gram for radium.

As Dorsey described [5], the accuracy of these measurements required the radium-226 to be in equilibrium with the radon-222 and other daughters, because the electroscope responded primarily to energetic gamma rays from the radium C daughter (bismuth-214). In addition, the accuracy of the electroscope measurements required a standard source configuration, an effective filter for the soft gamma rays from radium B (lead-214), and a reproducible fixed distance from the measurement plane of the electroscope. However, qualitative measurements for plant quality control could be obtained in instances such as those described by Hughes [2]: “Examination of the ore through crystallization; examination of the ore, examinations of the solutions containing radium through crystallization process and final determination of the radium salts.” The fidelity of such measurements would depend on working standards of microgram quantities of radium in laboratory glassware and reproducible protocols for making product comparisons, as described in the Hess and Damon paper in 1922 [22].

In addition, the electroscope could also be adapted to measure ionization of a gaseous sample of radon-222. The Lind electroscope was equipped with a chamber with two stopcocks [35]. After the gas was introduced into the chamber, the air ionization would be caused by alpha and beta particles as well as gamma rays from the radon-222 and its daughters. Lind and colleagues from the USBM used this method for assessing the radium content of ores as part of their efforts to support the U.S. radium industry. Constance Torrey at NBS developed a method for radium emanation (radon-222) measurements in 1923 with a German-made electroscope from Spindler-Hoyer [36]^3^3 Report of the Director of Bureau of Standards, 1923 (Constance Torrey page 86): In March the measurement of emanation with the new Spindler and Hoyer electroscope was commenced. By making possible the measurement of smaller preparations than is possible by the gamma-ray method, this method opens the way to production of small radioactive standards in solution for the use of scientific laboratories. The method also makes possible the more accurate tests of radioactive ore samples, a few of which have been included under the above-mentioned miscellaneous tests but have thus far been tested by the alpha-ray method only [36].. Albin von Sochocky and his business partner George Willis invented and marketed a similar design—the Sochocky-Willis Radioscope from Palo Company in New York—that was intended for measurements of radon from small samples [6, 37]. They provided a standard solution with which to calibrate the instrument.

## 4 The Victims, Their Assessments, and Their Diagnoses

As the RLMC ramped up production in 1917 to meet the military demands, they employed as many as 250 young women as dial painters at the peak of their activities [10]. The workers, some as young as 15 years old, received good wages at the time for semiskilled labor, although most were employed there only for a few years. Grace Fryer’s case was typical. She worked at the Orange, NJ, plant from 1917 to 1920, at which point she left for another job. In 1924, she visited a local dentist, Theodor Blum. He was dismayed by the severe dental problems and the atrophy of her jaw. He had never seen anything like it and immediately suspected a connection to her work with the lip-pointing paint brushes dipped in radium paint in her previous employment. To achieve the desired precision when painting small watches, the women were encouraged to draw their brush through their mouth (*i.e.*, “lip-point”) to obtain a finer point, meaning they would inadvertently ingest some of the radium-containing paint. Soon, other dial painters presented with similar dental problems and the condition that Blum had identified as “radium jaw.” The growing awareness in the local community had two consequences: (1) Public health officials, led by County Medical Examiner Dr. Harrison Martland and Dr. Alice Hamilton, a public health champion from Harvard University, began enquiries into exposures of the dial painters at what was now U.S. Radium Corporation (USRC); and (2) the company, sensing negative publicity and possible liabilities, began to contract for studies by scientific experts and physicians to demonstrate that the small number of employees affected were victims of other diseases [8, 9].

Table 1 here summarizes key information about 11 sets of radiological measurements taken on victims of radium poisoning in New Jersey in the period 1925 to 1929.

**Table 1:** Radioactivity measurements on radium workers, 1925–1929.

Dates	Individuals	Investigators	Instrument Types	Notes	References
Early 1925	Grace Fryer	Harrison Martland	Electroscope	Breath analysis	Berry papers Reel 1, p. 48 [2, 7]
June 5, 1925	Edwin Leman	Harrison Martland Albin von Sochocky Howard Barker	Electroscope	Autopsy	Moore, p. 127 [11] Mullner, p. 68 [10] Martland, p. 70 [7]
June 16, 1925	Sarah Maillefer	Harrison Martland Albin von Sochocky	Lind Electroscope	External 18 inches (46 cm) above chest Breath analysis	Moore, pp. 129, 130 [11] Mullner, p. 69 [7, 10]
Summer 1925	Marguerite Carlough	Harrison Martland	Lind Electroscope	Breath analysis	Moore, p. 141 [7, 11]
October 15, 1927	Amelia (Mollie) Maggia	Armin St. George Charles Norris Alexander Gettler Ralph Muller		Autopsy	Mullner, p. 48 [10, 38]
November 1927	Grace Fryer + 4	Elizabeth Hughes	Lind Electroscope	Breath analysis	Berry papers [2]
April 22, 1928	Grace Fryer + 4	Frederick Flinn Herman Schlundt Howard Barker	Electroscope	External 2–3 ft (0.6–0.9 m) from patient	Moore, p. 209 [11] Berry papers [2]
April 22, 1928	Grace Fryer + 4	Alexander Gettler Elizabeth Hughes	Electroscope	External	Martland, p. 76 [7]
April 1928	Grace Fryer + 4	Elizabeth Hughes	Phosphorescent screen	Breath analysis	Martland, p. 77 [7]
November 1928	Grace Fryer + 4	Gioacchino Failla Herman Schlundt Howard Barker	Electroscope	External; confirmed radioactivity in all 5 individuals	Moore, p. 242 [11] Martland, p. 76 [7]
October 1929	Mae Cubberley Canfield	Elizabeth Hughes	Lind Electroscope	Breath analysis	Clark, p. 138 [9]

## 5 The 1925 Cases: Edwin Leman, Marguerite Carlough, and Sarah Maillefer

The first case that brought focused attention to radium poisoning was not a dial painter, but the USRC company’s chemist Edwin Leman (1888–1925), who died of aplastic anemia on June 5, 1925 [7, 10, 11]. Leman, with a Ph.D. in chemistry from the University of Chicago, had worked with radium for 14 years, with the last four years at USRC. The local medical examiner, Harrison Martland, suspected radium poisoning and reached out to von Sochocky to assist with an autopsy that revealed elevated radium in several organs. von Sochocky suggested they look at some of the dial painters, who were experiencing problems as well, and a few days later, they turned to the sisters Marguerite Carlough and Sarah Maillefer [11]. Sarah was seriously ill and was examined first. Martland and von Sochocky devised two methods for measuring the radium in the bodies of living patients. For external whole-body gamma-ray measurements of radioactivity, they placed a leaf electroscope at 18 inches (46 cm) above the chest. It was understood that this measurement of the torso would not be the same geometry as a point source of radium, but this was a compromise between being too close and losing signal strength at greater distance. They also sampled expired air (breath) for radon-222 using what they believed to be the first usage of the Lind electroscope with a sample chamber for the expired air. In both the gamma-ray measurement and the expired air, they measured the drift rate in terms of divisions per minute as Dorsey had described (Appendix B) [5]. Immediately following Sarah’s death on June 18, 1925, Martland conducted an autopsy [7, 10]. In this instance, Martland reduced the bone samples to ash to allow a more quantitative estimate of the radium content.

## 6 The 1927 Cases: Chancery Court of New Jersey

In May of 1927, a young New Jersey attorney, Raymond Berry, filed a suit in New Jersey against the company USRC on behalf of Grace Fryer and four coworkers: Katherine Schaub, Quinta née Maggia Macdonald, Albina née Maggia Larice, and Edna Hussman [10, 11]. Quinta and Albina Maggia had worked at RLMC for a couple of years before leaving to be married. Their older sister Amelia (known as Mollie) had continued working at USRC until her death in 1922. After her sisters joined in the lawsuit against USRC, Berry convinced the family to allow Mollie’s body to be exhumed to see if she had also been a victim of radium poisoning.

The body was exhumed on October 25, 1927, and was found to contain 48.4 μg of radium, an amount that was 500 times what would be later set as a limit for human body burden. The autopsy was performed by Drs. Armin St. George and George Norris, and several other medical doctors [38]. Alexander O. Gettler, a Ph.D. chemist who served as medical examiner for the City of New York, and his colleague, New York University chemist Ralph Muller, collected bone and tissue samples for radioactivity measurements at Bellevue Hospital. They used an electroscopic method to show radioactivity in the bones and also obtained images using photographic film wrapped around the bones for extended exposures.

Elizabeth Hughes was on hand as an observer at the autopsy [11]. In her *curriculum vitae*, Hughes listed her occupation in 1925–1927 as a physical chemist working for von Sochocky [1]. As she had two small children during this time, she may have simply been available as an assistant as needed for his consulting practice. von Sochocky’s health was deteriorating between 1925 and 1928 [7, 10]. Martland had diagnosed him as radioactive in 1925, and he had spent time in Colorado trying to recuperate. Hughes listed her employer in 1928–1930 as Raymond Berry, attorney [1].

Weeks after Amelia Maggia’s autopsy in November 1927, Hughes made measurements of expired air (radon-222) for the five dial painters in the case [2]. Thus, in late 1927, Hughes made the transition from scientific assistant to Albin von Sochocky to expert consultant for the attorney Raymond Berry.

The technical details of Hughes’s measurements and her commentary on the measurements of the other scientists are contained in the testimony of the Chancery Court from April 25–26, 1928. The Raymond Berry papers of the National Consumers League are on microfilm rolls at the Library of Congress [2]. Hughes testimony came in several segments: First, she was questioned by the plaintiffs’ attorney, Raymond Berry, and then cross-examined by the USRC company’s lawyer, Edward Markley. This was followed by redirect and further cross-examination by the lawyers. Occasionally, Judge John Backes, of the Chancery Court, would ask Hughes to clarify technical points to allow him to understand the significance of the science.

In the initial round of questioning from Berry, Hughes listed her college education and her professional employment at the NBS and at the Electroscope Laboratory in New Jersey (Appendix A). She said she had been a physicist at NBS with an annual salary of $1440. She informed the court on the nature of alpha, beta, and gamma radiations and the differences between radium (meaning radium-226) and mesothorium (radium-228). Berry asked her to describe the expired air test that she performed on the five women dial painters in some detail.

“The set up was as follows: the Lind Electroscope was used; that is an instrument used to detect radioactivity. The patient breathes through a series of driers, a series of five bottles of calcium chloride, to remove the moisture, glass wool to remove particles of dust, two bottles of sulphuric acid, and another to remove – another of glass wool to remove any further dust. The patient breathes through these bottles, which were connected to one outlet of the ionization chamber the electroscope, and [at] the other outlet a suction pump was used to help draw the breath through. A period of about five minutes was given for breathing, then readings were taken on the instrument. The rate of the discharge of the leaf of the electroscope was twice – in all cases, twice, and in some cases more, the normal drift of the leaf.” ^4^4 The preceding is a transcription of the testimony by the court recorder, and there are enough misspellings and typeovers to indicate the court had some problem following the technical jargon of this witness.

Hughes elaborated that this indicated the presence of radioactivity. When Berry asked if this was a qualitative or quantitative test, she indicated that it was qualitative; the trace amount of radioactivity in the expired air was too small to determine quantitively.

Hughes provided details on her awareness of the dangers of radium exposure and protective measures that were in place for the scientists who chose to use them. She said, “At the Bureau of Standards we all had this put before us…… Rutherford was our handbook at the Bureau of Standards and he brings this all out in his own book on radioactivity.” She described Dorsey’s experience with burns to his hands and the protective measures (shields for the torso and forceps to handle sources) he put in place at the NBS to limit exposures.^5^5 Quote from Sven Kjaer visit to U.S. Bureau of Standards, April 13, 1925 [39]: “...radiations produce aplastic anemia, suppression of menstruation in female workers and cause sterility. Precautions were taken here, as in other establishments where considerable radium is handled, to protect workers against burns by use of forceps for handling the tubes and to protect them against radiation by use of lead screens, lead containers and distance from source of radiation. Blood counts of workers were taken periodically and any found affected were given vacations until recovered, or transferred to other work.” Berry proceeded to question Hughes about the scientific reputations of Victor Hess and Albin von Sochocky. She was impressed with Victor Hess but objected to referring to von Sochocky as a radium physicist; she said he was president of the company, but when pressed further she testified, “I should say he was a chemist, primarily. Manufacturer. Knew how to manufacture the radium—chemistry and physics of radium are two entirely separate things.” She went on to express respect for Hess and reported that she had read some of his papers written during the time she worked for him. It probably would have helped her testimony if she had mentioned that she coauthored a paper with him on radium measurements that was published in *Physical Review* [22].

When it was the USRC lawyer Markley’s turn to cross-examine Hughes, he attacked her credentials as a physicist. He had her admit that she was a housewife with small children and had not worked in the laboratory for the past five years. She had not done any formal graduate work in physics but continued to read scientific papers pertaining to radium. She said she had always been in contact with Dr. Dorsey at NBS. He questioned her if she knew of the work of the Pittsburgh chemist Charles Viol and physician Frederick Proescher. She had read some, but not all, of their papers in the journal *Radium* and noted that the journal was produced by their employer the Standard Chemical Company. Markley saved his sharpest questioning for Hughes on her “qualitative indications of radioactivity” from the expired air tests of the five patients. She admitted that this was the first time she had administered the expired air tests to individuals, and that the tests had to be done very carefully. He asked for more details on how the drift rate was measured. He pointed out that moisture in the chamber would result in a faster drift of the leaf. Hughes replied that she already reported that she used a series of driers to eliminate the moisture and that normal persons had been sampled as well to provide the baseline drift rate.

Markley went to some effort to establish that the amount that she had measured was an insignificant amount and that she could not state the limit of detection (LOD) of the instrument. He did not refer to an LOD, but that was the gist of his questioning. Markley kept asking for a quantitative estimate of the amount of radioactivity that could be measured in the expired air tests with the electroscope. Hughes insisted that it was a qualitative test that showed twice the drift rate of the leaf. However, at some point, she relented and said, according to the court transcript, “Thousandth of a millimicrogram.” It is not clear whether Hughes was stating a limit of detection of radium of a nanogram or a picogram, but this was the result that Markley was looking for, that is, that it was a tiny amount.^6^6 Lind’s 1915 description of the electroscope suggests a detection limit of the order of nanograms [35], while the limit described by Robley Evans’ later work—perhaps with more refined detectors—is an order of magnitude lower [32]. After a recess of the court, Markley returned to this line of questioning to cast doubts on the validity of the expired air tests.

## 7 The USRC Electroscopic Measurements of the Dial Painters

In further questioning during the Chancery Court hearing, Markley then asked Hughes if she was aware of the radioactivity measurements of the five women that had been conducted the past Sunday (April 22, 1928) [2]. The USRC required the women to undergo a compulsory examination by Frederick Flinn from the Institute of Public Health at Columbia University (a consultant to the company), Herman Schlundt, a chemist from the University of Missouri, and Howard Barker, the USRC vice president and chief physicist [11]. They performed whole-body radium measurements with an external electroscope. They found no evidence of radium contamination. The court also allowed the chemist Gettler and the physicist Hughes to make their own measurements with the electroscope. They reported that at least one of the women tested positive for radioactivity [7]. Finally, Martland reported on another set of measurements by Hughes that had been suggested by von Sochocky [7].

“Shortly after this examination, at the suggestion of von Sochocky, Mrs. Hughes prepared screens of pure phosphorescent zinc sulphide uncontaminated by radioactive substances. After these had been examined in the dark room for scintillations, with negative results, each girl blew her expired air over these screens beneath the microscope. Scintillation from the presence of alpha particles was easily demonstrated in all five cases, proving beyond a doubt that they had deposits of radioactive substances in their bodies, which were giving off emanation.”

Hughes’ time in the Chancery Court of New Jersey finally concluded with Markley still questioning the significance of the results and Berry reinforcing the consistency and the competence of the witness. Hughes reiterated that her positive measurements of the expired air were “qualitative” indicators of radioactivity. She noted that, in performing the tests, she had received assistance in the laboratory and set up by Dr. Harrison Martland, the medical examiner. This is important, in that it explains why she had access to the apparatus to conduct the expired air tests. With her experience in chemistry, she could easily construct the gas-handling system to scrub the radon, and she was experienced in the use of the leaf electroscope. However, she needed someone to provide the expensive apparatus. It might have been the first time that Hughes had administered the expired air tests, but Martland had taken breath samples from Grace Fryer in early 1925.^7^7 “Late in 1924, Dr. Harrison Martland of Newark, requested Miss Fryer to come to see him. He asked her to make several visits, which she did. On the occasion of her visits, Dr. Martland took several samples of her blood. He did not tell her why he was doing this, except that it was for experimental purposes. During these visits Miss Fryer breathed into a tube of an apparatus set up in Dr. Martland’s office. In July of 1925, Dr. Martland informed Miss Fryer that as a result of a thorough investigation which he had made, her system showed the presence of radioactive substances and that her illnesses had all been caused by radioactive substances in her body.” (p. 48 in Raymond Berry papers) [2]. In closing, she mentioned that she had *received a few points by letter from Dr. Dorsey at the Bureau of Standards*.

Following the April 1928 trial, the USRC asked for a delay because their experts would be on travel abroad during the summer. There was widespread condemnation of the company in the press for attempting to delay a court judgement until the women died. Marie Curie, Norman Thomas, a socialist candidate for president, and Walter Lippman, a renowned journalist, all called for justice for the five women [10, 11]. On June 4, 1928, federal Judge William Clark, acting as a negotiator between the claimants and the USRC, had the two parties reach a settlement that provided substantial funds to the seriously ill women. The company did not admit liability for their illnesses. Berry later pointed out that Judge Clark had some financial interests in the company [11].

A few months later, in November of 1928, the company made one more attempt to prove that the five women were not contaminated with radium. This time the company scientists, Barker and Schlundt, were assisted in the electroscopic measurements by Gioacchino Failla, a pioneer in medical physics at New York’s Memorial Hospital (now Sloan Kettering Memorial Hospital) [7, 11, 40]. He pronounced that all five patients were radioactive.

Berry was also successful in his last case against the USRC on behalf of the dial painter Mae Cubberley Canfield. In October of 1929, Hughes took a breath sample from Canfield for radon measurement with the Lind electroscope [9]. This case was also settled out of court in 1930. Hughes received $800, her 10% share of the settlement. This was the last lawsuit against the USRC involving Berry and Hughes, as the attorney had agreed as part of the settlement to no longer represent dial painters with claims against the company.

## Part II: Leon F. Curtiss and Constance Torrey

## 8 The National Radium Conference, December 1928, Washington, D.C.

**Fig. 3:**
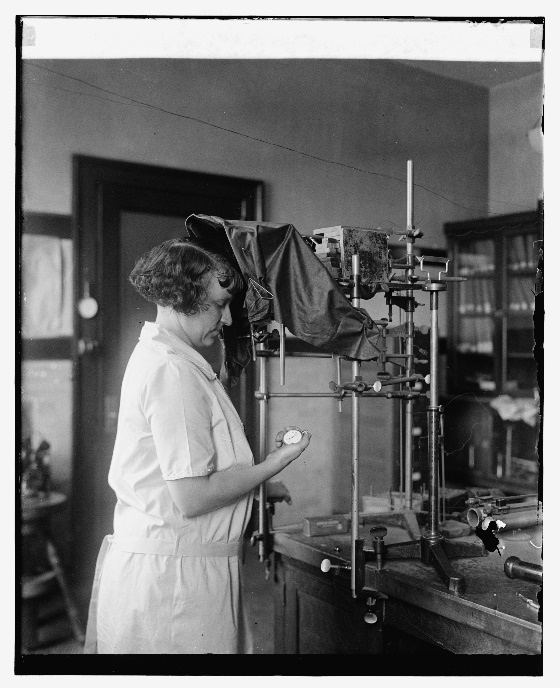
Constance Torrey shown in 1925 with stopwatch and NBS electroscope [4].

**Fig. 4:**
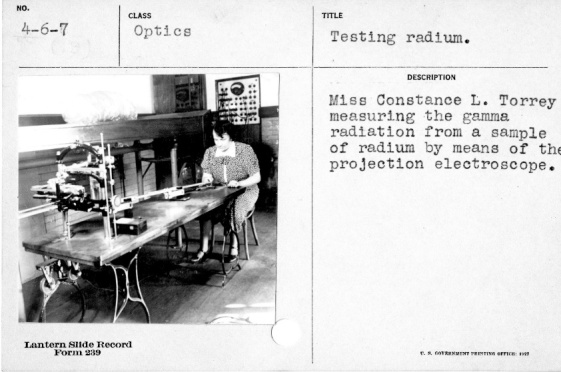
Constance Torrey with projection electroscope in 1927 [4].

**Fig. 5:**
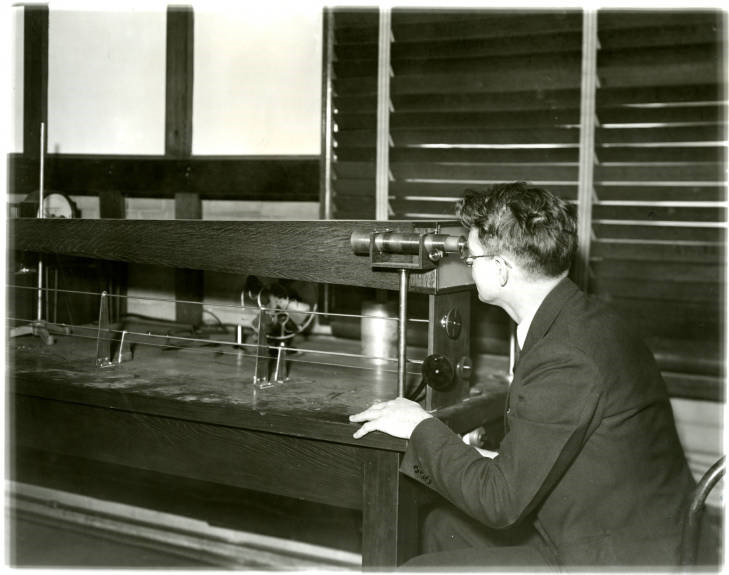
Leon Francis Curtiss with projection electroscope circa 1928 [4].

In 1928, Leon F. Curtiss (1895–1983), the lead physicist for radioactivity measurements in the Atomic Physics, Radium and X-Ray Section at the Bureau of Standards, was drawn into the deliberations on radium health effects. In late 1926, Curtiss had built a projection electroscope such that radium source measurements carried out by the physicist Constance Torrey could be made with the operator at some distance from the sources under test [41]. Figure 3 shows Torrey in 1925 holding a stopwatch to record the drift rate on a leaf electroscope that is probably the same one that Mary Brower was using two years prior (Fig. 2). Figure 4 shows Torrey with the projection electroscope built by Curtiss, and Curtiss himself is shown in Fig. 5 with an improved version of the system.

On December 20, 1928, the USPHS held a “voluntary” one-day conference on radium [9,10], although as reported by Clark, USPHS was reluctant to confront the lucrative radium industry on a matter of occupational workplace exposures. The USPHS was being pressured by Alice Hamilton from Harvard University and from the general public that had followed the newspaper accounts of the dial painters. This voluntary conference was attended by leaders of the radium industry and many of the experts previously mentioned, including Frederick Flinn, Harrison Martland, and Alice Hamilton. U.S. federal agencies were invited as well: the Department of Labor, Bureau of Mines, and NBS. The Bureau of Mines, which had employed both academic chemists Samuel Lind and Herman Schlundt, was a strong proponent of industry. Leon Curtiss and Lauriston Sale Taylor (1902–2004) were there to represent the NBS. Mullner reported that two committees were to be formed: one on establishing codes on safeguards and best protective practices, and a second on workforce regulations [10]. One assumes that scientists would have been on the first committee and labor specialists and industrial hygienists would have been on the second.

The U.S. Surgeon General placed these advisory committees under the USPHS Office of Industrial Hygiene and Sanitation. Their deliberations were underwhelming to the public health advocates [9]. In 1933, the USPHS reports were finally published [42] in four parts in the *Journal of Industrial Hygiene*, a journal that Alice Hamilton had established in 1919. Some of the preliminary findings had already been released; for example, in 1931, a paper by Harrison Martland [43] and a preliminary summary of the investigations by James Leake were published [44]. Parts I and III of the lengthy USPHS report *The Health Aspects of Radium Dial Painting* provide a detailed overview of the scientific aspects of the radioactivity measurements of the dial painters [42]. The reports were, of course, too late to be of much help to the early dial painters, and the authors benefited from the past decade of research developments in instruments and techniques. The USPHS reports followed the guidance and recommendations of an expert advisory committee of physical scientists, which included:

• Dr. Leon F. Curtiss, physicist, U.S. Bureau of Standards, Washington, D.C.,
• Professor William Duane, professor of biophysics, Harvard University, Boston, MA,
• Dr. Gioacchino Failla, physicist, Memorial Hospital, New York, New York,
• Dr. Oliver H. Gish, chief of the Section on Terrestrial Electricity, Carnegie Institute of Terrestrial Magnetism, Washington, D.C.,
• Professor Samuel C. Lind, director of the School of Chemistry, University of Minnesota, Minneapolis, MN,
• Dr. Harrison Martland, chief medical examiner, Essex County, NJ, and
• Professor Herman Schlundt, professor of physical chemistry, University of Missouri, Columbia, MO.

Curtiss, the youngest of the seven, was listed first alphabetically. Six of these seven made significant contributions to radium and radon measurements over three decades from 1910 to 1940. Most of them spent time studying radioactivity with the masters: with the Curies in Paris, with Rutherford in Cambridge, and with Stefan Meyer in Vienna as shown in Table 2.

The chemists, Lind and Schlundt, had both spent time with the USBM in Denver and Washington, D.C., and were experts on radium chemistry, and both provided consulting services to the radium industry. The physicists, Duane and Failla, were leaders in the emerging field of radiotherapy using both radium needles and radon seeds, in which radon-222 was obtained daily from gram quantities of radium-226 in sulfuric acid solutions. Duane had mastered the extraction techniques while working with Marie Curie, and Failla became expert in producing encapsulated radon seeds for therapy [40]. Failla had recently returned to New York from a sabbatical year with Marie Curie. Oliver Gish was probably included on the committee because of his early work with Millikan on cosmic rays [49]. His colleague, Serge Korff at the Carnegie Institute, was soon collaborating with Curtiss at NBS on cosmic-ray investigations using radiosondes launched in weather balloons [26]. Harrison Martland was a critical member of the advisory committee because of his extensive medical examinations and involvement with radioactivity measurements for the dial painters over the past three years [48]. Curtiss was selected because NBS maintained the national standards for radium, and he had published papers on electroscopic measurements of radium sources [41].

The “advisory committee” to the USPHS designed a protocol to investigate the extent of radium poisoning in the luminous dial industry. The USPHS investigation, led by Senior Physicist James Ives and Assistant Physicist Fred L. Knowles, began in June 1929 and concluded in March 1930 [42]. The scope and findings were published in Part I in September 1933, and the measurements of radioactivity in workers were published in Part III in November 1933. Hundreds of dial painters and radium workers (both men and women, but mostly younger women) and controls were included. Those with exposure to radium and

**Table 2:** Selected group of scientists engaged in radium measurements in the United States in the period 1910–1930.

Individual	Discipline	Affiliation in 1928	European Research Experience	Principal Contributions to Radium and Radon Measurements	References
Leon Francis Curtiss 1895–1983	Physicist	Bureau of Standards	Rutherford, Cavendish Laboratory 1922–1926	Radium standards Electroscope Radon pump	[4]
Gioacchino Failla 1891–1961	Physicist	Memorial Hospital, NY	Curie, Institute du Radium 1925	Radium and radon seeds	[40]
Samuel Colville Lind 1879–1965	Chemist	University of Minnesota	Meyer, Radium Research Institute 1911 Curie, Institute du Radium 1910	Radium chemistry Lind electroscope	[35, 45]
Herman Schlundt 1869–1937	Chemist	University of Missouri	Rutherford, Cavendish Laboratory 1921	Radium-228 chemistry Radium physiology	[18, 19, 20]
William Duane 1872–1935	Physicist	Harvard University	Curies, Sorbonne Paris 1905 Curie, Institut du Radium 1906–1912	Radon generator Medical physics	[34,46]
Robley Dunglison Evans 1907–1995	Physicist Graduate student	California Institute of Technology	—	Radium and radon measurements	[47]
Albin Sabin von Sochocky 1883–1925	Physician	Deceased Died of radium poisoning 1928	Curies, Sorbonne Paris 1906	Radium chemistry Sochocky-Willis electroscope	[6, 14, 37]
Harrison Martland 1883–1954	Physician Pathologist	Medical Examiner New Jersey	—	Radium physiology Patient measurements	[48]

mesothorium were divided into two groups: those who had worked *before* January 1, 1927 (Group B), and those who began their work with radium *after* January 1, 1927 (Group A). It was postulated that the early workers had possibly engaged in lip-pointing of their brushes with the luminous paint, and that this practice had been discouraged after that time. Individuals were subjected to blood tests, physical examinations, and electroscopic exams with both gamma-ray determinations and “exhaled air for presence of radon and thoron.” These authors referred to “exhaled air” rather than “expired air,” the term used in the 1928 New Jersey trial. The authors explicitly acknowledged the assistance of Leon Curtiss, Samuel Lind, and Gioacchino Failla for calibrations of instruments and assistance with the field measurements.

Part I of the report contained 16 findings of the investigation and 28 recommendations for minimizing the hazards of radium in dial painting. Several of the findings are interesting from a radiation-health-physics perspective. They found the results of the gamma-ray tests and the radon and thoron tests to be generally consistent with one another.^8^8 Evans noted later 1937 that there was a reason that the emanation measurements were lower than those obtained by external electroscope methods [50]. In both Groups A and B, the red cell count and hemoglobin tended to be lower than in the controls, although the supporting data set was not included in the paper. The Group A (after 1927) cohort also had ingested radium; the largest body burden in Group A was 3.5 µg of radium, and the largest body burden in Group B (before 1927) was 11.3 µg of radium; this provided a clear indication of the need for additional protective measures. In both groups, the radium accumulation in the body was associated with the length of radium exposure. There was no indication of the presence of radium in the control group.

**Fig. 6:**
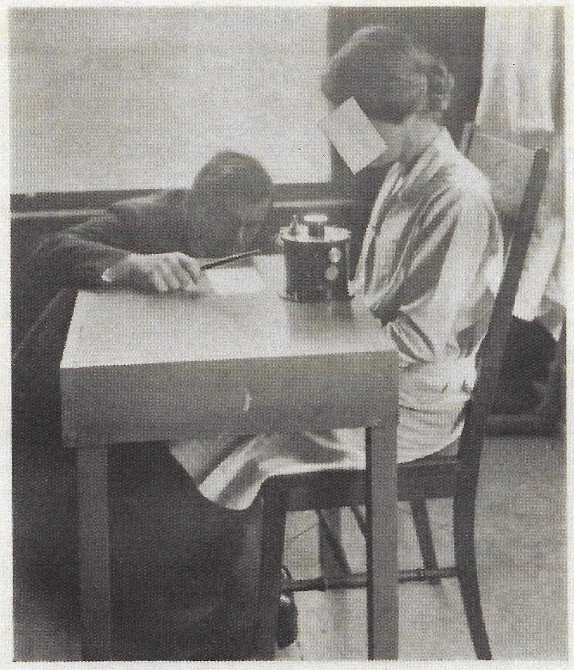
Radium measurement by the gamma-ray method (USPHS) 1933 [42].

Part III of the report by the USPHS physicists provided more detail on the methods with which the radioactivity measurements in the workers were carried out. The gamma-ray determinations were made with a Wulf bifilar (Hess type) electroscope [51, 52, 53]. The electroscope placement in a standard geometry with a subject is shown here in Fig. 6 [42].

Failla was credited for providing the known-activity radon-222 seeds inserted into a cadaver to calibrate the electroscope for the gamma-ray measurements. The probable error of an individual gamma-ray determination was taken as 0.3 µg. All subject controls measured less than 0.8 µg.

## 9 Radium Protection Standards and the National Council on Radiation Protection and Measurements (NCRP)

Clark’s book mentions the halting steps of the public health community to take up the case of the dial painters [9]. It is interesting that, at the same time that the USPHS began an investigation, there was a parallel effort organized by U.S. medical physicists and radiologists. This group was to become the National Council on Radiation Protection and Measurements (NCRP). The initial genesis of the NCRP in 1929 came from the push by the international radiology community to establish radiation protection guidelines for workers using X-ray systems for diagnosis and therapy [54, 55]. However, radiation-protection standards for radium were also included in their mandate. The first of the NCRP committees in the 1930s included two of the experts from the USPHS advisory committee [56, 57]. Failla represented the Radiological Society of North America (RSNA), and Curtiss served as secretary of the NBS Handbook 23 [57].

There was one expert missing from the USPHS and NCRP deliberations in 1929, because Robley D. Evans (1907–1995) was only a 22 year old student at the time [47]. Evans began his studies of radium poisoning as a graduate student under Robert Millikan at the California Institute of Technology and continued as a young physics professor at the Massachusetts Institute of Technology [32, 58]. Evans’s papers in 1932–1937 [59 - 62] filled in the blanks left by these other leading scientists on the limits of detection of radon and thoron (radon-222 and radon-220) using electroscopic methods. It is not that the more experienced physicists could not have optimized the equipment and established baselines; they simply had other research objectives (*e.g.*, cosmic rays, industry contracts, medical physics) that took precedence. Evans’s 1932 paper in *Physical Review* described the accurate determination of low levels of radon and thoron [59], and his 1933 review paper in the *American Journal of Public Health* [60] gave a complete overview of published work on radium poisoning. A critical contribution was his 1937 paper in the *Journal of Roentgenology and Radium Therapy* [50] that allowed him to correlate the amount of radium in the body with the exhaled radon. This required him to build the first whole-body counter to measure the gamma rays. A brief summary of the technical details of state-of-the-art of measurements of radium poisoning in 1920–1940 is given in Appendix E.

The early published federal reports are often referred to as “NBS standards.” That is because the original committee reports chaired by Lauriston Taylor and Leon Curtiss were published by the Department of Commerce as NBS Handbook 18 (in 1934 [56]), NBS Handbook 23 (in 1938 [57]), and NBS Handbook 27 (in 1941 [63]). The latter report was able to incorporate the results from Evans’s research over the past decade. Based on this body of work from the 1930s, the advisory committee for NBS Handbook 27 proposed a limit at 0.1 µg for the body burden of radium-226. The nine men who prepared the report agreed to set a tolerance level for residual radium in the body such that they would be perfectly comfortable if their wives and daughters were the subjects [32, 55, 58]. They were all in agreement, but that rationale was omitted from the published NBS guidance.

## 10 Summary and Conclusions

By the time the USPHS investigations were published in November 1933 and the radiation protection experts summarized their recommendations in NBS Handbook 27 in May of 1941 [63], it was understood that the principal hazards from radium were gamma rays from external sources, inhalation of radon and radium on dust particles, and ingestion of radium through the mouth by unsanitary practices. In combination or separately, these insults could lead to aplastic anemia, necrosis of the jaw, and osteosarcomas and other maladies. The collective action on the part of the public health, medical physics, and worker protection communities led to best practices that reduced but did not eliminate the hazards from the radium industry. The lip-pointing with radium was effectively discontinued after 1926, but there were late effects among dial painters. The radium dial painting continued, with more stringent protective measures in place, until alternative radionuclidic sources (hydrogen-3 and promethium-147) were identified. Lang reported in *The New Yorker* in 1959 [64] on the measurements of many of the dial painters who had been monitored by the U.S. Atomic Energy Commission into the late 1950s. A summary of the U.S. Department of Energy studies (a continuation of the Atomic Energy Commission work) was published by Rowland [65] from the Argonne National Laboratory in 1995. Three decades later, the Million Person Study by the NCRP led Martinez and coworkers to a comprehensive review of the dosimetry of the early radium workers using modern methods [12].

In 1921, the mission of the Radium Section at NBS was to carry out measurements and develop standards of radium to support applications in industry and defense. Over the following two decades, the NBS staff was slowly engaged to provide national measurements and standards for other purposes, including medicine, public health, and the environment. During this 20 year period (Appendix D), the “Radium Section” was subsumed into a larger group in the Optics Division [4]. There was no section leader or division chief who could direct staff to look into emerging applications. However, from the late 1920s, Leon Curtiss began to develop the tools for radionuclide metrology and radiometry that could be applied to the luminous dial industry [66–69]. In 1940, Curtiss was named the first Section Chief for Radioactivity in a new Atomic and Radiation Physics Division. In May of 1941, the Department of Commerce published NBS Handbook 27 on *Safe Handling of Radioactive Luminous Compound* [63]. NBS was pressed to take on the federal leadership for the project by the representative from the U.S. Navy, Captain (later Admiral) Charles S. Stephenson. Curtiss chaired the advisory committee that prepared this handbook, a group that included Evans, Martland, Failla, and Flinn, along with representatives from the U.S. Departments of Labor and Navy and the radium industry. This federal guidance on radiation protection included the limit of 0.1 µg for the body burden of radium. The work of Curtiss’ section during and after World War II included thousands of measurements of radon for ore and breath analyses, techniques for measurement of the brightness of luminous dials, and national standards for radiation protection in the luminous dial industry. As Evans and others have reported [10, 32, 58, 64], the timely benefit of the testing done on radium dial painters in the 1920s and early 1930s was to establish maximum concentrations—or body burdens—for internal contamination with alpha-particle-emitting radionuclides. This research was then applied to radiation-protection standards for plutonium-239 produced in the Manhattan Project.

## 11 Epilogue

Elizabeth Damon Hughes was an unsung heroine in the saga of the radium dial painters. She was a young scientist of about the same age as the dial painters, and she made the critical measurements that helped them win their day in court. It is not clear why she decided to return to the NBS in 1931, but the office she returned to was on the same hallway with Leon Curtiss and Constance Torrey. It is possible that she assisted Dr. Harrison Martland and the physicists at NBS in the electroscope calibrations for that last campaign of radium and radon testing for the control study of the dial painters in 1929–1930.

## 12 Appendix A. Elizabeth Damon Hughes (1898–1988) [1, 2, 4, 22, 70]

1898 Elizabeth Elmore Damon, born September 3 in Minden, LA
1915 Elizabeth Elmore Damon, freshman in applied science, University of Rhode Island
1919 Baccalaureate degree, University of Vermont
1919–1920 Laboratory assistant, U.S. Bureau of Standards
1920–1922 Physicist, U.S. Radium Corporation (August 1920–September 1922)
1922 Coauthor of paper in *Physical Review* with Victor Hess (July) [22]
1922 Married Edward Henry Hughes in Kingston, RI (October 13, 1922) [70]
1925–1927 Physical chemist for A. S. von Sochocky, founder and former president of USRC
1927 November 1927, collected breath samples from five dial painters
1928–1930 Radium expert for Raymond H. Berry, Attorney
1928 April 25–26, testified in Chancery Court, NJ, on radon breath testing [2]
1929 Agreed to measure expired air (radon) for Mae Cubberley Canfield, October 11
1931 Returned to NBS. Phone Book listings in 1933:
Hughes, Room 309 East Building
Mohler (Chief) Room 312 E
Leon Curtiss, Constance Torrey, Leroy Stockman, Room 317 E
1934 Located in Room 333 Industrial Building
1959 Chemist in NBS Physical Chemistry Section 5.8
1961 Retired from NBS
1988 Died September 24, 1988

## 13 Appendix B. Use of the NBS Electroscope

Noah Ernest Dorsey gave a concise description of the electrometric methods used for relative measurements of radium in his 1921 book *Physics of Radioactivity* [5].

“The simple electroscope consists of a metal case within which, and near its center, is supported in a vertical position a well-insulated metal strip to the top of which is attached a narrow strip of thin foil, preferably of gold leaf. This strip of foil is usually spoken of as the leaf. The strip of metal and the leaf constitute the insulated system of the electroscope. When the insulated system is electrically charged by a suitable switch passing through the wall of the case, the leaf is repelled by the strip, and is deflected from its normal, vertical position. In opposite sides of the case are windows through which the position of the leaf can be observed. Such observation is usually made by means of a microscope having in its eyepiece a ruled scale. When intended for gamma ray measurements, the electroscope should be carefully screened on all sides except one with lead at least one inch thick, so that the air in the electroscope will be protected from scattered radiations that would otherwise enter it. For the same reason, the windows should be as small as is conveniently possible. The ionizing radiation of which the intensity is to be measured enter[s] the electroscope through the unscreened side. In order to minimize the effect of the absorption of the radiation by the wall of the container and by the salt itself, the measurements should be based upon the hard, penetrating radiation emitted by radium-C. For this reason it is desirable that the radiations entering the electroscope be filtered through lead at least 15 mm thick so that very little of the soft gamma radiation from radium-B enters the electroscope. When everything is ready, all radium preparations are placed at such distances and so screened that they produce as small an ionization in the electroscope as possible—the preparations to be compared must be so placed that they produce only a negligible ionization. The insulated system is then charged and insulated, and the time required for the image of the leaf to move over a few divisions near the middle of the scale in the microscope is determined. From this, the rate of drift of the leaf, in divisions per second, when there is no radium near the electroscope, is determined. This is called the natural, or the blank, drift. It results from imperfect insulation and slight residual ionization of the air. Then the tube under test is placed in a suitable position, and the time required for the leaf to drift over a certain portion of the scale is determined. If the blank drift is subtracted from the rate of drift observed when the tube under test is in position, the difference will be the rate of drift due to the radiation from the tube; this is known as the corrected drift. The tube under test is now removed to its former position where it does not affect the electroscope, and the standard tube is placed in exactly the same position previously occupied by the tube under test. Its corrected drift is determined in exactly the same way as was that for the tube under test. The ratio of the two corrected drifts is equal to the ratio of the intensities of the two radiations; which, in turn, is equal to the ratio of the amounts of radium-C that are contained in the two tubes, provided that the absorption of the radiations by the walls of the two tubes is the same in both cases. Knowing the amount of radium-C in the standard, the amount in the tube under test can now be computed at once.” —Noah Ernest Dorsey

## 14 Appendix C. Albin Sabin von Sochocky

1882 Born February 22 in Lany, Ukraine, then part of the Austrian-Hungarian Empire [6]
1900 University of Lviv, Ukraine, studied physics, chemistry, and medicine [10]
1905 University of Moscow, medical degree [6, 10]
1905 Course work in Vienna (with Stefan Meyer?), Austria, and Prague, Czech Republic (then part of the Austrian-Hungarian Empire), and Dresden, Germany [6, 10]
1905 Met with the Curies in Paris [6, 10]
1906 Began a practice of medicine in New York [10]
1913 Discovered luminous paint (*Undark*) in New York City (Moore, p. 16 [11], Mullner, p. 42 [10])
1914 Founded RLMC in Newark, NJ, with George S. Willis (Moore, p. 17 [11], Mullner, p. 42 [10])
1917 Hired Florence E. Wall to work in plant physics laboratory (Clark, p. 17 [9])
1920 Established the Electroscope Laboratory to limit radiation to instruments (Clark, p. 17 [9])
1921 On January 21, pushed out as president of the RLMC by Treasurer Arthur Roeder.
Company reorganized as U.S. Radium Corporation with Roeder as president
1921 In January, profiled in popular article in *American Magazine* [14]
1925 1925–1927, employed Elizabeth Hughes as technical assistant [1]
1925 Harrison Martland diagnosed him as radioactive (Clark, p. 127 [9], Martland, p. 74 [7])
1925 June 7, measured radium in bones of Edwin Leman (died June 5, 1925) (Clark, p. 102 [9])
1925 In June, examined Marguerite Carlough (with Martland), whole body + air (radon) [7]
1925 In February, realized nature of disease in painters (Moore, p. 128 [11])
1928 April 27, testified in Chancery Court, NJ (Moore, p. 219 [11])
1928 Died November 14, 1928 [6]
15. Appendix D. Time Line of NBS Radium Measurements and Dial Painters
1913 U.S. Bureau of Standards (N. E. Dorsey) begins to establish standards for radium [4].
1920 N. E. Dorsey describes workload for radium measurements and lists staff [4].
1921 Report is published by British expert committee on hazards of radium for workers.
1923 U.S. Public Health Service study staff of NBS Radium Section [29].
1927 November, Elizabeth E. Hughes obtains breath samples from five plaintiffs [2].
1928 April 25–26, Elizabeth E. Hughes testifies in Chancery Court of New Jersey
for five radium dial painters [2].
1928 Leon Curtiss publishes papers on a radon pump [67] and a projection electroscope [41].
1928 U.S. Surgeon General holds public health conference and selects an advisory committee
(Leon Curtiss, Samuel Lind, William Duane, Gioacchino Failla, and others) [10].
1933 Four-part U.S. Public Health Service study is published in *Journal of Industrial Hygiene* [42].
1934 Bureau of Standards Handbook No. 18 is produced on protection from radium [56].
1938 Briggs *et al.* publish radium protection guidelines in journal *Radiology* [71].
1938 NBS Handbook No. 23 supersedes Handbook No. 18 on radium protection [57].
1941 NBS Handbook No. 27, *Safe Handling of Radioactive Luminous Compound*, is published [59].
1942 Curtiss paper on prevention and control of hazards in dial painting industry is published [68].
1943 Curtiss and Davis paper on pulse ion chamber for radium ore and breath samples is published [69].
1946 NBS annual report lists 400 air samples (likely ore and breath samples) [4].
1949 NBS annual report lists 729 radon breath samples [4].
1950 NBS annual report lists 900 radon breath samples [4].
1950 Leroy Stockman participates in local NBC TV show on NBS radon testing laboratory [4].
16. Appendix E. State-of-the-Art Measurements for Radium Poisoning 1920–1940

There were three methods available in the early 1920s to ascertain whether a subject was a victim of radium poisoning. For living subjects, one could measure the external gamma rays from radium-226 and progeny with an electroscope, or one could measure the radon-222 and progeny in exhaled breath with an electroscope fitted with a sample collection chamber. The third (and posthumous) method was to perform an autopsy on bones or other organs. The radium-226 extraction from this process could then be assayed using a radon-222 emanation technique, with the same type of electroscope as that used for the exhaled breath. The end point for all three of these methods was to determine the body burden of radium in the individual. The nominal values of the body burdens for the radium workers varied from 1 µg to 180 µg depending on their type of work and length of exposure [61]. The sources of uncertainty in the measured values were different for the three methods. Before considering the uncertainties encountered in the early measurements, it is useful to refer to a common set of physiochemical parameters that would apply to typical, in this case female, subjects as shown in Table 3.

**Table 3:** Nominal physiochemical parameters for a radium dial painter in 1920.

Parameters—Young Woman	Nominal Values
Mass	50 kg
Bone mass	2 kg
Mass of humerus bone	100 g
Radium body burden	1 µg
Steady-state radon radioactivity	1 µCi
Expiration rate	16 breaths per minute
Minute volume expiration	5.6 L/min

## 16.1 Autopsy Methods

The first autopsies were performed on the chemist Edwin Leman and on the dial painter Sarah Maillefer in 1925 (Table 1). Martland also performed autopsies on Marguerite Carlough and Eleanor Eckert [7, 11]. In Leman’s case, tissue samples revealed radioactivity in the lungs, spleen, and bones. There was suspicion that he had inhaled radioactive dust in the radium plant. In the dial painter Sarah Maillefer’s case, the radium was found in her spleen, liver, and bones [10]. In both cases, they assayed the extracted radium by measuring the radon emanation with an electroscope in the USRC Electroscope Laboratory. Two years later, Amelia (Mollie) Maggia’s body was exhumed, and an autopsy was performed that indicated extensive radium poisoning [38]. These 1929 measurements by the chemists Gettler and Muller and the physician St. George in the *Archives of Pathology* are among the first reliable total body measurements by autopsy. They made quantitative measurements on four soft-tissue organs (lung, liver, spleen, and brain), and all were found to be radioactive. In addition, they made measurements on eight bone segments, including skull, femur, vertebrae, and jaw. Ninety-nine percent of the radioactivity was in the skeleton. They calibrated the Lind electroscope with a known mass of carnotite (Lind had recommended using pitchblend for this purpose [35]). Their result for the body burden was 48.4 µg of radium. The three significant figures in this result are probably not warranted, as they made several assumptions in summing up the fractions of activity measured in the tissue samples, and the “radium content was from about 22 percent of the entire body weight only.” However, this left little doubt that the victim died of radium poisoning.

## 16.2 Whole-Body Gamma-Ray Methods

The whole-body gamma-ray measurements began as early as 1925 with Sarah Maillefer shortly before her death [10, 11]. The later subjects were seated facing a charged electroscope, and the drift rate of the leaf, corrected for the normal drift, was compared with the drift rate for a known sealed radium source [42]. Of course, the geometry of a subject containing systemic radium poisoning did not approximate a point source. In the 1928 measurements by Flinn and Schlundt, the latter attempted to calibrate the system by placing two 5 µg radium sources in his vest pockets and positioning himself facing the electroscope [7]. Flinn then held the electroscope 0.6 m to 0.9 m (2 ft to 3 ft) away from the subjects with a table screening part of their bodies [11]. He astutely realized that, using 1/*r*^2^, he could place the subjects far enough away from the electroscope to demonstrate that no radium was present. The Chancery Court allowed Alexander Gettler, the chemist from Bellevue Hospital in New York, and Elizabeth Hughes to make electroscopic measurements as well [7]. They reported at least one of the women tested positive by the electroscopic method [7]. When Failla from Memorial Hospital made measurements with the USRC scientists, he pronounced that Grace Fryer and the other four plaintiffs were all radioactive [11].

In Part III of the USPHS report, the investigators had developed a much more formal protocol for gamma-ray measurements of the radium workers. The subjects were seated at a specially designed table (see Fig. 6) with the electroscope at the edge near the subject and the subject’s thighs directly under the table. The Wulf bifilar electroscopes were calibrated in advance at NBS and were charged several hours or longer prior to the measurements. A calibration factor was established in terms of the drift rate for 1 µg of radium “distributed throughout the body.” This calibration factor was established by Failla by placing calibrated radon-222 seeds in a cadaver positioned in the test geometry. Body-burden values for the subjects tested varied from 1.0 µg to 11.5 µg radium (results included those contaminated with radium-228 as well as those contaminated with radium-226). The highest control reading was 0.8 µg, and the “probable error” for individual determinations was 0.3 µg radium.

Robley Evans in 1937 described the first of the modern whole-body radiation detectors [50]. He included the physics to deal directly with the geometry of the subject and the self-absorption of the radium C (bimuth-214) gamma rays in tissue. The subject was positioned on a table at the same height as the detector with the body bent into a 1 m arc such that the source-detector distance always approximated 1 m. He introduced a small tube counter to replace the bulky and less sensitive electroscope. The tube counter was 2 cm diameter by 12 cm long, at 6.5 cm Hg pressure and operated at 170 V. The tube counters were claimed to be from 10 to 100 times more sensitive than the best electroscopes. Radium sources calibrated against the International Radium Standard were placed at different positions around the arc to determine the detector response. The final equation to relate the subject measured response to the radium in the body took into account the distance from the subject and the mean thickness of the body. The system would detect 1 µg of radium at 1 m distance. Results were described for a radium dial painter (53 kg) who had painted watch dials from 1922 to 1929. Seven years later, she was found to contain 9.7 ± 0.5 µg radium.

## 16.3 Exhaled Air (Radon/Thoron) Methods

Martland might have been one of the first to measure radon in exhaled breath when he measured Grace Fryer in early 1925. However, as Evans pointed out in his 1935 paper [61], measurements of radon alpha particles with a special chamber on an electroscope was a valuable tool for many applications, including analyses of ores, fractions from radium separations, and validation of radium “standard” solutions, as well as its uses for biological specimens of bone ash. Lind and von Sochocky had both produced commercial versions of this type of electroscope by the early 1920s [35, 37]. Early NIST archives mention radium emanation [4] but do not describe such measurements until the director’s annual report from 1923 [36]:

“In March the measurement of emanation with the new Spindler and Hoyer electroscope was commenced. By making possible the measurement of smaller preparations than is possible by the gamma-ray method, this method opens the way to production of small radioactive standards in solution for the use of scientific laboratories. The method also makes possible the more accurate tests of radioactive ore samples, a few of which have been included under the above-mentioned miscellaneous tests but have thus far been tested by the alpha-ray method only.”

The “small radioactive standards in solution” would have been prepared by serial dilution of milligram quantities of radium previously calibrated by the gamma-ray method. The availability of nanogram per milliliter (ng mL^−1^) standard solutions in 1923 allowed scientific laboratories to accurately assay radon-222 at the nanocurie level. The USPHS physicists in the 1929–1930 study [42] used four electroscopes that had been calibrated by Curtiss and Torrey at NBS. The four chambers allowed them to measure four women simultaneously. The rate of ionization in the 2.7 L chamber increased rapidly for the first 20 min and reached a maximum in about 3 h. The average value for the calibration coefficient from the NBS’s calibrations was 14.84 × 10^−9^ Ci of radon for one division-per-second drift rate on the leaf electroscope. Rather than the four-figure accuracy on the calibration coefficient, it would be interesting to see the highest and lowest values of the four measurements.

The second important calibration coefficient in the 1929 study was “2.097 × 10^−6^.” Evans [50] used the same formalism and gave the value of 2.097 × 10^−6^ as the number of curies of radon-222 per second produced by the body in equilibrium with 1 g of radium-226. With these two calibration coefficients and a measured net drift rate for the electroscope, the investigators could calculate the body burden of radium (in grams):

*m* (*g*) = (*R* × *F_1_*)/ (*T_C_* × *F_2_*) (1)

where

*R* = net drift rate (divisions s^−1^),

*F_1_* = NBS calibration coefficient (Ci/divisions s^−1^),

*T_C_* = collection time for 2.7 L (s), and

*F_2_* = radon-222 emanation rate (Ci s^−1^ g^−1^ radium-226).

The breath samples were collected over a period of 10 min in a “Douglas bag” having a volume of 2 cubic feet (~0.06 m^3^). This collection rate corresponds to 56.6 L in 600 s. That gives a value of *T_C_* = 28.6 s for the 2.7 L in the ionization chamber. Measured drift rates and Eq. (1) were used to compute the body burdens for the radium workers and controls under test. The “probable error” in the values computed in this manner were estimated to be ± 0.3 µg of radium. A similar methodology was used for thoron (radon-220), but the uncertainties were greater due to the compressed sampling and measurement times for the 54.5 s half-life radionuclide.

As stated previously, the investigators in the USPHS study [42] did find a good correlation between the body burden estimates obtained by the gamma-ray method and the radon emanation method. Selected results are shown Table 4. Values were not included for subjects who tested positive for thoron and for those having less than 2 µg as measured by the gamma-ray method.

**Table 4:** Selected values from Table 4 in Ives, Knowles, and Britten, *Journal of Industrial Hygiene*, 1933 [42].

Subject Number	Radium, Gamma-Ray Method (µg)	Radium, Emanation Method (µg)
196	9.3	11.7
213	6.1	1.9
107	3.5	1.2
103	2.8	0.2
202	2.7	3.6
236	2.2	2.8
240	2.0	0.9
Mean value	4.1	3.2

The methods do appear to give consistent results for those workers who had a substantial body burden of radium. The results obtained by the emanation method are about a third less than those obtained by the gamma-ray method. Evans noted in his later papers that there are subject-to-subject differences in this ratio of radium measurements by the two methods depending on the time and manner in which the radium was ingested. His paper with Aub, Hempleman, and Martland [72] reported that, on average, about 55% of the radon produced in the body is exhaled in breath, although there is wide variation in this figure.

## References

[1] Sager EEH (1955) American Men of Science: A Biographical Directory, Vol. 1: Physical Sciences (The Science Press/R. R. Bowker Company, New York, NY), 9th Ed.

[2] Berry RH (1928) Raymond H. Berry Papers of the National Consumers League pertaining to case of Grace Fryer *et al. vs*. U.S. Radium Corporation. Microfilm Reel 1, Library of Congress, Washington, D.C.

[3] Cochrane RC (1966) Measures for Progress: A History of the National Bureau of Standards (U.S. Department of Commerce, Washington, D.C.). Available at https://nvlpubs.nist.gov/nistpubs/Legacy/MP/nbsmiscellaneouspub275.pdf

[4] National Institute of Standards and Technology (2021) Archives of the National Bureau of Standards (U.S. Department of Commerce, Washington, D.C.).

[5] Dorsey NE (1921) Physics of Radioactivity (Williams and Wilkins, Baltimore, MD). Available at https://babel.hathitrust.org/cgi/pt?id=nyp.33433066415948&view=1up&seq=7&skin=2021

[6] Wall FE (1976) Sabin Arnold von Sochocky: 1882–1928. American Chemists and Chemical Engineers, ed Miles WD (American Chemical Society, Washington, D.C.), 488 p.

[7] Martland HS (1929) Radium Poisoning. Occupational poisoning in the manufacture of luminous watch dials. *Monthly Labor Review**,* U.S. Bureau of Labor Statistics 28(6):62–95.

[8] Clark C (1993) Radium poisoning revealed: A case study in the history of industrial health reform. *Humboldt Journal of Social Relations* 19(1):73–116 (Department of Sociology, Humboldt State University, Arcata, CA). Available at https://www.jstor.org/stable/23262526

[9] Clark C (1997) Radium Girls: Women and Industrial Health Reform, 1910–1935 (University of North Carolina Press, Chapel Hill, NC). 11623540

[10] Mullner R (1999) Deadly Glow, The Radium Dial Painter Tragedy (American Public Health Association, Washington, D.C.), ISBN: 0-87553-245-4.

[11] Moore K (2017) Radium Girls: The Dark Story of America’s Shining Women (Sourcebooks, Inc., Naperville, IL).

[12] Martinez, NE, Jokisch DW, Dauer LT, Eckerman KF, Goans RE, Brockman JD, Tolmachev SY, Avtandilashvili M, Mumma MT, Boice JD, Leggett RW (2021), Radium dial workers: Back to the future. International Journal of Radiation Biology. Published online 26 April 2021. 10.1080/09553002.2021.1917785 PMC10563809 33900890

[13] Wall FE (1969) Early days of radioactivity in industry, part I. Chemistry 42(4):17–19. Available at https://semspub.epa.gov/work/02/55209.pdf

[14] Von Sochocky SA (1921) Can’t you find the keyhole? American Magazine, p 24.

[15] Landa ER (1987) Buried Treasure to Buried Waste: The Rise and Fall of the Radium Industry (Colorado School of Mines, Golden, CO). ISBN 13: 9780918062758

[16] Fulton County Tribune (1920) Gram of radium for New York State. *Fulton County Tribune*, August 13, 1920 (Wauseon, OH). Available at https://chroniclingamerica.loc.gov/lccn/sn87076552/1920-08-13/ed-1/seq-7/

[17] Lubenau JO, Landa ER (2019) Radium City: A History of America’s First Nuclear Industry (Senator John Heinz History Center, Pittsburgh, PA

[18] Schlundt H (1922) *Mesothorium*, (U.S. Department of the Interior, Bureau of Mines, Washington, D.C.), Technical Paper No. 265.

[19] Schlundt H (1931) The refining of mesothorium. Journal of Chemical Education 8(7):1267.

[20] Gibbons B (2013) Chronology: Herman Schlundt and radiation research at Missouri’s Pickard Hall. *Columbia Missourian*, July 15, 2013. Available at https://www.columbiamissourian.com/news/chronology-herman-schlundt-and-radiation-research-at-mu-s-pickard/article_ffd075bf-bc53-5f59-ad77-10c15ca16b7d.html

[21] Hess VF (1963) Victor Francis Hess. Current Biography Yearbook 1963, ed Moritz Ch (H. W. Wilson Company, New York, NY), pp 180–182. Available at https://www.mpi-hd.mpg.de/hfm/HESS/public/hessbio.html

[22] Hess VF, Damon EE (1922) Improvement in the determination of the radium content of low-grade radium-barium salts. Physical Review 20(1):59-64.

[23] Coursey BM, Colle R, Coursey JS (2002) Standards of radium-226: From Marie Curie to the International Committee for Radionuclide Metrology. Applied Radiation and Isotopes 56:5–13. Available at https://www.sciencedirect.com/science/article/pii/S0969804301001592 11839059 10.1016/s0969-8043(01)00159-2

[24] Foote P (1966) William F. Meggers Memorial Pamphlet, c. 1966. William F. Meggers biographical file, Archives of the National Institute of Standards and Technology (National Institute of Standards and Technology, Gaithersburg, MD). (Paul Foote quote regarding Marie & Irene Curie visit to NBS in May 1921.)

[25] Lescarboura AC (1921) A chat with Madame Curie. Scientific American, July 9, 1921, p 25. Available at https://www.scientificamerican.com/article/a-chat-with-madame-curie/

[26] Coursey BM (2020) History of atmospheric cosmic ray research at the National Bureau of Standards. Journal of Research of the National Institute of Standards and Technology 125:125001. 34900399 10.6028/jres.125.001 PMC8341379

[27] Rutherford E (1905) Radio-Activity (Cambridge University Press, Cambridge, U.K.), 2nd Ed., pp 216–217. Available at https://archive.org/details/radioactivity00ruthgoog/page/n7/mode/2up

[28] Rutherford E (1913) Radioactive Substances and Their Radiations (Cambridge University Press, Cambridge, U.K.).

[29] Williams RC (December 21, 1923) Preliminary note on observations made on physical condition of persons engaged in measuring radium preparations. *U.S.* Public Health Reports 38(51):3007.19314909

[30] Washington Evening Star (1924) Mary Brower. *Washington Evening Star*, June 8, 1924.

[31] Stewart E (1929) Radium poisoning. *Monthly Labor Review**,* U.S. Bureau of Labor Statistics 28(6):20–61.

[32] Evans RD (1980) Origins of standards for internal emitters. *Health Physics, A Backward Glance**,* eds Kathren RL, Ziemer PL (Pergamon Press, New York, NY), pp 141–157.

[33] Curie M (1923) Pierre Curie with Autobiographical Notes (Macmillan, New York), English Ed., translated by Charlotte and Vernon Kellogg.

[34] Duane W (1915) On the extraction and purification of radium emanation. Physical Review 5:311–314.

[35] Lind SC (1915) Practical methods for the determination of radium: I. Interchangeable electroscope and its use. Journal of Industrial and Engineering Chemistry 7(5):406–410.

[36] National Bureau of Standards (1923) Annual Report of the Director of the Bureau of Standards to the Secretary of Commerce for the Fiscal Year Ended June 30, 1923 (U.S. Department of Commerce, Washington, D.C.). U.S. Department of Commerce Miscellaneous Publication 53. Available at https://www.govinfo.gov/content/pkg/GOVPUB-C13-75443eca5356f3c796e590228d830981/pdf/GOVPUB-C13-75443eca5356f3c796e590228d830981.pdf

[37] Modern Hospital (1916) Advertisement for Sochocky-Willis radioscope from the Palo Co. of New York. *Modern Hospital* 6:234. Available at https://books.google.com/books?id=iTYhAQAAMAAJ&pg=RA3-PA234-IA36&lpg=RA3-PA234-IA36&dq=Palo+Company+New+York+Electroscopes&source=bl&ots=5oVBbpkBdK&sig=ACfU3U1UCbxt4Q0oSS1liooVNVwQUQ-4NA&hl=en&sa=X&ved=2ahUKEwjExq7Z5ZLyAhUZMlkFHeo7BscQ6AEwBnoECAwQAw#v=onepage&q=Palo%20Company%20New%20York%20Electroscopes&f=false

[38] St. George AV, Gettler AO, Muller RH (1929) Radioactive substances in a body five years after death. Archives of Pathology 7:397–405.

[39] Kjaer S (1925) Occupational Diseases—Radium Necrosis. Summary of Field Work (Argonne National Laboratory, Argonne, IL), Box 118, Argonne National Laboratory Archives, U.S. Radium Collection, Sven Kjaer Papers, Center for Human Radiobiology, April 13, 1925.

[40] Atomic Heritage Foundation (2021) National Museum of Nuclear Science and History profile on Gioacchino Failla Available at https://www.atomicheritage.org/profile/gioacchino-failla

[41] Curtiss LF (1928) A projection electroscope for standardizing radium preparations. *Journal of Optical Society of America* and *Review of Scientific Instruments* 16(5):363–366.

[42] Schwartz L, Knowles FL, Britten RH, Thompson LR (1933) Health aspects of radium dial painting I: Scope and findings, *Journal of Industrial Hygiene* 15:362; Bloomfield JJ, Knowles FL (1933) Health aspects of radium dial painting II: Occupational environment. *Journal of Industrial Hygiene* 15:368; Ives JE, Knowles FL, Britten RH (1933) Health aspects of radium dial painting III: Measurements of radioactivity in workers. *Journal of Industrial Hygiene* 15:433; Schwartz L, Makepeace FC, Dean HT (1933) Health aspects of radium dial painting IV: Medical and dental phases. Journal of Industrial Hygiene 15:447.

[43] Martland HS (1931) The occurrence of malignancy in radioactive persons. American Journal of Cancer 15(4):2435–2516. Available at https://cancerres.aacrjournals.org/content/15/4/2435.article-info

[44] Leake JP (1932) Radium poisoning. Journal of the American Medical Association 98(13):1077–1080.

[45] Laidler KJ (1998) *Samuel Colville Lind: 1879–1965* (National Academy of Sciences, Washington, D.C.), Biographical Memoirs. Available at http://www.nasonline.org/publications/biographical-memoirs/memoir-pdfs/lind-samuel.pdf

[46] Bridgman PW (1936) *A Biographical Memoir of William Duane: 1872–1935.* (National Academy of Sciences, Washington, D.C.), Biographical Memoirs. Available at http://www.nasonline.org/publications/biographical-memoirs/memoir-pdfs/duane-william.pdf

[47] Michigan State University (2021) Profile on Robley D. Evans (Michigan State University, East Lansing, MI). Available at https://ehs.msu.edu/lab-clinic/rad/hist-figures/evans.html

[48] Rutgers Radiation Research History (2021) Martland H. Archives in the George F. Smith Library (Rutgers University, New Brunswick, NJ). Available at https://njms.rutgers.edu/departments/division_radiation/history_pub.php

[49] Kargon RH (2020) The Rise of Robert Millikan: Portrait of a Life in American Science (Plunkett Lake Press, Lexington, MA). https://www.google.com/books/edition/The_Rise_of_Robert_Millikan_Portrait_of/trAHEAAAQBAJ?hl=en&gbpv=1&dq=oliver+gish+millikan+cosmic+rays&pg=PT110&printsec=frontcover

[50] Evans RD (1937) Radium poisoning. II. The quantitative determination of the radium content and radium elimination rate of Living persons. American Journal of Roentgenology and Radium Therapy 37:368–378.

[51] Wulf T (1909) Ein neues Elektrometer für statische Ladungen. *Physikalisch Zeitschrift* 8:246 (1907); 10:211.

[52] Hess VF (1913) Űber Neuerungen und Erfahrungen an den Radiummessungen nach der γ-Strahlenmethode: I. Eine modification des Wulfschen Strahlungsapparates. Physikalisch Zeitschrift 14:1135.

[53] Hess VF, Schmidt W (1918) Uber die verteilung radioakiver Gase in der frein Atmosphäre. Physikalisch Zeitschrift 19:109–114.

[54] Taylor LS (1981) X-Ray Measurements and Protection: 1913–1964. (National Bureau of Standards, Washington, D.C.), NBS Special Publication 625. Available at https://nvlpubs.nist.gov/nistpubs/Legacy/SP/nbsspecialpublication625.pdf

[55] Taylor LS (1981) Organization for Radiation Protection: The Operations of ICRP and NCRP: 1928–1974 (U.S. Department of Energy, Washington, D.C.), Document DOE/TIC 10124.

[56] National Bureau of Standards (1934) Radium Protection for Amounts up to 300 Milligrams (U.S. Department of Commerce, Washington, D.C.), Bureau of Standards Handbook No. 18. Available at https://orau.org/health-physics-museum/files/library/nbs/nbs-18-radium-protection.pdf

[57] National Bureau of Standards (1938) Radium Protection (U.S. Department of Commerce, Washington, D.C.), National Bureau of Standards Handbook No. 23 (supersedes Handbook No. 18). Available at https://nvlpubs.nist.gov/nistpubs/Legacy/hb/nbshandbook23.pdf

[58] Evans RD (1981) Inception of standards for internal emitters, radon and radium. Health Physics 41:437–448. 7026502

[59] National Bureau of Standards (1941) Safe Handling of Radioactive Luminous Compound (U.S. Department of Commerce, Washington, D.C.), National Bureau of Standards Handbook No. 27. Available at https://nvlpubs.nist.gov/nistpubs/Legacy/hb/nbshandbook27.pdf

[60] Evans RD (1932) Determination of small quantities of radon and thoron. Physical Review 30:1014.

[61] Evans RD (1933) Radium poisoning: A review of present knowledge. American Journal of Public Health 23:1017–1023. 18013838 10.2105/ajph.23.10.1017-b PMC1558329

[62] Evans RD (1935) Apparatus for the determination of minute quantities of radium, radon and thoron in solids, liquids and gases. Review of Scientific Instruments*,* 6:99.

[63] Evans RD, Mugele RA (1936) Increased gamma‐ray sensitivity of tube counters and the measurement of the thorium content of ordinary materials. Review of Scientific Instruments 7:441.

[64] Lang D (1959) A most valuable accident. *New Yorker Magazine*, May 2, 1959, p 49. Available at https://www.newyorker.com/magazine/1959/05/02/a-most-valuable-accident

[65] Rowland RE (1994) Radium in Humans: A Review of U.S. Studies. (Argonne National Laboratory, Argonne, IL), ANL/ER-3. Available at https://publications.anl.gov/anlpubs/1994/11/16311.pdf

[66] Curtiss LF (1927) Pyrex as a container for radium solution. Nature 120:406.

[67] Curtiss LF (1928) A radon pump. *Journal of Optical Society of America* and *Review of Scientific Instruments*, 17(1):77–80.

[68] Curtiss LF (1942) Prevention and control of hazards in the radium dial painting industry. Journal of Industrial Hygiene and Toxicology 24:131–141.

[69] Curtiss LF, Davis FJ (1943) A counting method for the determination of small amounts of radium and radon. Journal of Research of the National Bureau of Standards 31:181. Available at https://nvlpubs.nist.gov/nistpubs/jres/31/jresv31n3p181_A1b.pdf

[70] The Poultney Journal (1922) Hughes-Damon Wedding. The Poultney Journal, October 13, 1922 (Poultney, VT).

[71] Briggs L (1938) Radium Protection. Radiology 31(4):481–490.

[72] Aub JC, Evans RD, Hempleman LH, Martland HS (1952) The late effects of internally-deposited radioactive materials in man. Medicine 31(3):221–329. 12982323 10.1097/00005792-195209000-00001

